# Celebrating 10 Years of the HIMSS-SIIM Enterprise Imaging Community and Enterprise Imaging Informatics

**DOI:** 10.1007/s10278-024-01141-7

**Published:** 2024-06-10

**Authors:** Christopher J. Roth, Cheryl A. Petersilge, Dawn Cram, Kim Garriott, Lou Lannum, Cheryl K. Carey, Nikki Medina, Tammy Kwiatkoski, James T. Whitfill, Alexander J. Towbin

**Affiliations:** 1 Department of Radiology, Duke University, Durham, NC USA; 2 University of Pittsburgh School of Medicine, Pittsburgh, PA USA; 3 Vidagos, Chagrin Falls, OH USA; 4 Department of Research and Development, PaxeraHealth, Newton, MA USA; 5 NetApp, Indianapolis, IN USA; 6 Lannum & Associates, Cleveland, OH USA; 7 Society for Imaging Informatics in Medicine, Leesburg, VA USA; 8 Healthcare Information and Management Systems Society, Chicago, IL USA; 9 HonorHealth, Scottsdale, AZ USA; 10 University of Arizona College of Medicine - Phoenix, Phoenix, AZ USA; 11 Department of Radiology, Cincinnati Children’s Hospital, University of Cincinnati College of Medicine, Cincinnati, OH USA

**Keywords:** Enterprise imaging, Standards, HIMSS-SIIM white papers, Imaging informatics

## Abstract

In response to the growing recognition of enterprise imaging as a critical component of healthcare’s digital transformation, in 2014, the Healthcare Information and Management Systems Society (HIMSS) and the Society for Imaging Informatics in Medicine (SIIM) signed a Memorandum of Understanding to form the HIMSS-SIIM Enterprise Imaging Community (HSEIC). At the time of the agreement, the two organizations decided to collaborate to lead enterprise imaging development, advancement, and adoption. This paper celebrates the past 10 years of the HSEIC’s thought leadership, industry partnerships, and impact while also looking ahead to identify enterprise imaging challenges to solve in the next decade.

## Background

The 2009 American Recovery and Reinvestment Act (ARRA) drove US hospitals to install electronic health records (EHR) [1]. Capabilities not previously available to many clinical specialties were made accessible via the EHR. Still, EHR workflow design and implementation (and later optimization) revealed insufficient image and video management workflows for some use cases, including medical photography, point-of-care ultrasound, and scope procedures. At the time, few organizations had considered the vast horizontal of imaging across medical specialties, the importance of making images and video available in a centralized manner, and the relevance to care continuity. Instead, they purchased specialty vertical imaging archives and viewers to address specialty-specific gaps.

Changing technology and new legislation caused HIMSS, SIIM, and the healthcare information technology landscape to grow and evolve rapidly. Leaders of SIIM recognized that a partnership with HIMSS would provide a platform for imaging informatics professionals (IIPs) and non-imaging informatics professionals to work together to address enterprise imaging workflow gaps. Seeing an opportunity, society leadership for HIMSS and SIIM in 2014 established the HIMSS-SIIM Enterprise Imaging Community (HSEIC) to advance enterprise imaging strategies across healthcare organizations. In the first 10 years of this partnership, the HSEIC has advanced the field of enterprise imaging, helping to develop awareness, new workflows, and advocacy for industry change.

## Major Community Achievements

HSEIC’s major achievements are its educational outputs, the incorporation of enterprise imaging into the Digital Imaging Adoption Model (DIAM), community development, and ongoing efforts to advance enterprise imaging across all healthcare organizations through its impact on technology development.

### Educational Outputs

**Fig. 1:**
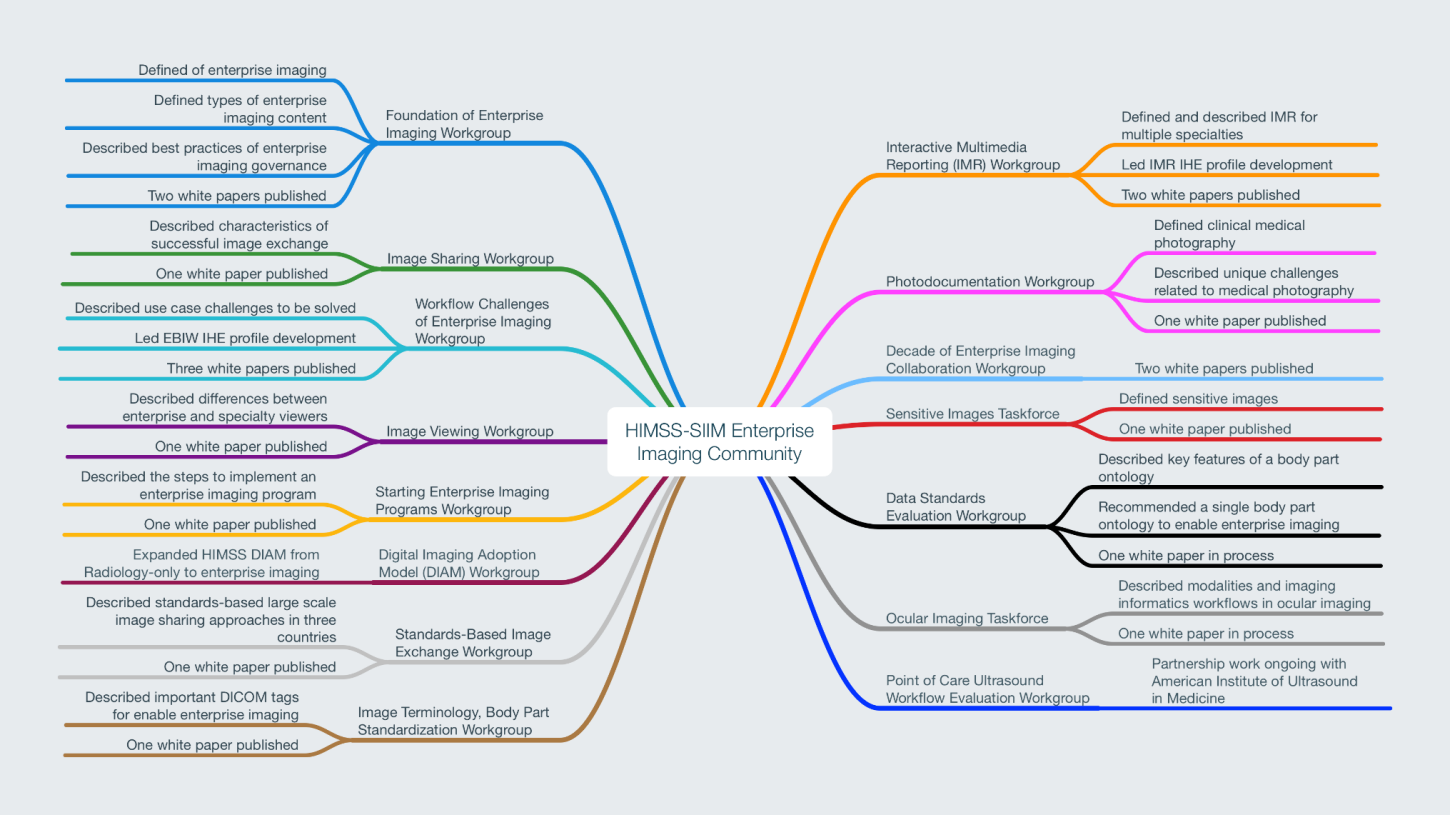
Workgroups, taskforces, and results of the HIMSS-SIIM Enterprise Imaging Community

**Fig. 2:**
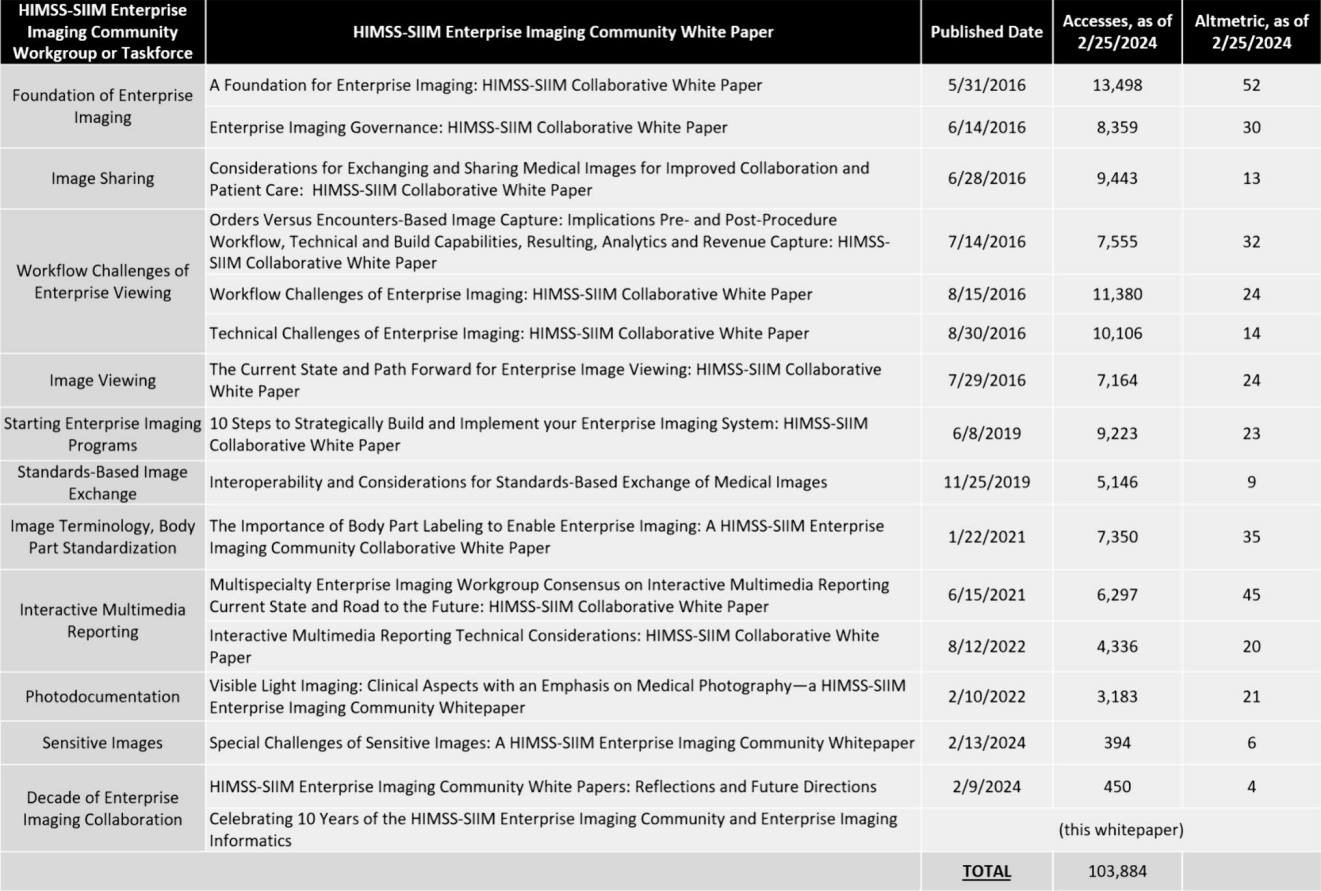
Open access HIMSS-SIIM Enterprise Imaging Community white papers with originating workgroup or taskforce, publication date, number of accesses, and Altmetrics

Driving thought leadership through white papers, workgroups, and regular HSEIC communications, “enterprise imaging” has become common parlance in hospitals, healthcare news media, and industry partners. Fifteen HSEIC workgroups and taskforces have been commissioned in all, detailing the current state, best practice, and future opportunities for several important enterprise imaging administrative and clinical functions, such as governance, ideal orders-based and encounters-based workflow, image exchange, and image viewing through a series of influential white papers (Fig. 1). Including this paper, the HSEIC has produced 16 open-access white papers that have been downloaded over 100,000 times, shaping industry standards and influencing enterprise imaging implementations across the globe (Fig. 2) [2–16]. The HIMSS Annual Conference and SIIM Annual Meeting have together hosted more than 150 educational sessions centered on enterprise imaging since 2016. HIMSS and SIIM have also presented over 100 enterprise imaging meetups, webinars, and podcasts.

HSEIC workgroups and taskforces are groups of volunteers of varying roles and experiences dedicated to collaboratively approaching complex problems that typically do not have one single, simple path forward. Constructive disagreements on multifaceted issues have surfaced, as hospitals employ different approaches to meet similar imaging needs. The richness of the discussions, disagreements, and questions led to more informed participants and insightful whitepapers. For example, the Foundation of Enterprise Imaging workgroup recognized several imperfect methods to segment multimedia content. While content could be broken into familiar clinical specialty silos (cardiology, obstetrics, radiology, surgery, others), the lines would have been too blurry, as a given image may be captured, interpreted, or consumed by many specialties. Categorizing content into DICOM and non-DICOM was considered. Eventually, the group decided to focus on clinical relevance more than data format and transfer mechanism, as it was unclear what standard formats would be most common years into the future for some clinical content. As a result, the Foundations workgroup did not deeply probe this DICOM/non-DICOM categorization. After several meetings discussing the nature of enterprise imaging content, the workgroup determined that categorizing content as diagnostic, procedural, evidentiary imaging, and/or image-based clinical reports would ultimately best illustrate the spectrum of enterprise imaging content. In the end, the discussion across imaging and non-imaging providers, industry partners, and IIPs to describe a nuanced, largely uncharted space led to a more refined classification and early enterprise imaging cultural growth.

### Digital Imaging Adoption Model

HIMSS Analytics™ has helped hospital systems measure the maturity of their informatics strategy and implementation since launching the Electronic Medical Record Adoption Model (EMRAM) in 2005. In 2016, HIMSS Analytics™ collaborated with the European Society of Radiology (ESR) to launch the DIAM, although this first DIAM iteration incorporated radiology only. In partnership with the HSEIC and the European Society of Medical Imaging Informatics (EUSOMII), HIMSS expanded the DIAM in 2019 to encompass enterprise imaging. Today, the DIAM helps organizations objectively measure their maturity across many areas of enterprise imaging capabilities, including image management, governance, workflow, decision support, patient engagement, and analytics. As many organizations recognize the best practices described by the HIMSS adoption models as directional and aspirational, the DIAM has become an unbiased and informed source guiding the development of their enterprise imaging strategic plan.

### Community Development

Today, the HSEIC comprises over 1225 members, including providers, IIPs, nurses, data scientists, vendor partners, and other occupations. Regular newsletters, webinars, online roundtables, and in-person meetups provide networking opportunities for members, which connect members across provider and industry organizations to solve local challenges, share best practices, and develop new solutions. As the HSEIC has grown, it has developed partnerships with other specialty societies to help address challenges pertinent to each imaging specialty. Examples include the HSEIC’s work with the American Institute of Ultrasound in Medicine (AIUM) to advance point-of-care ultrasound workflows and the Digital Pathology Association (DPA) to advance pathology workflows. There are numerous points of entry into the HSEIC for interested imaging informaticists of all personas, expertise, and roles, including signups on the SIIM website, HIMSS website, and at HSEIC Annual Meeting Meetups [17, 18].

### Advancing Enterprise Imaging in Practice

Driven by workgroup outputs, HSEIC members recognized that technology gaps led to clumsy clinical workflows. They sought to address these gaps by collaborating with Integrating the Healthcare Enterprise (IHE), a heavily vendor volunteer-led initiative jointly sponsored by HIMSS and the Radiological Society of North America (RSNA) with a mission towards standards-based interoperability [19]. Successfully developed interoperability profiles with HSEIC participation include encounter-based imaging workflow (EBIW), interactive multimedia reports (IMR), and integrated reporting applications (IRA) [20–22]. While these profiles were built within the Radiology IHE Domain, they were purposefully written to be inclusive of other specialties and imaging workflows.

## Future Challenges

**Fig. 3:**
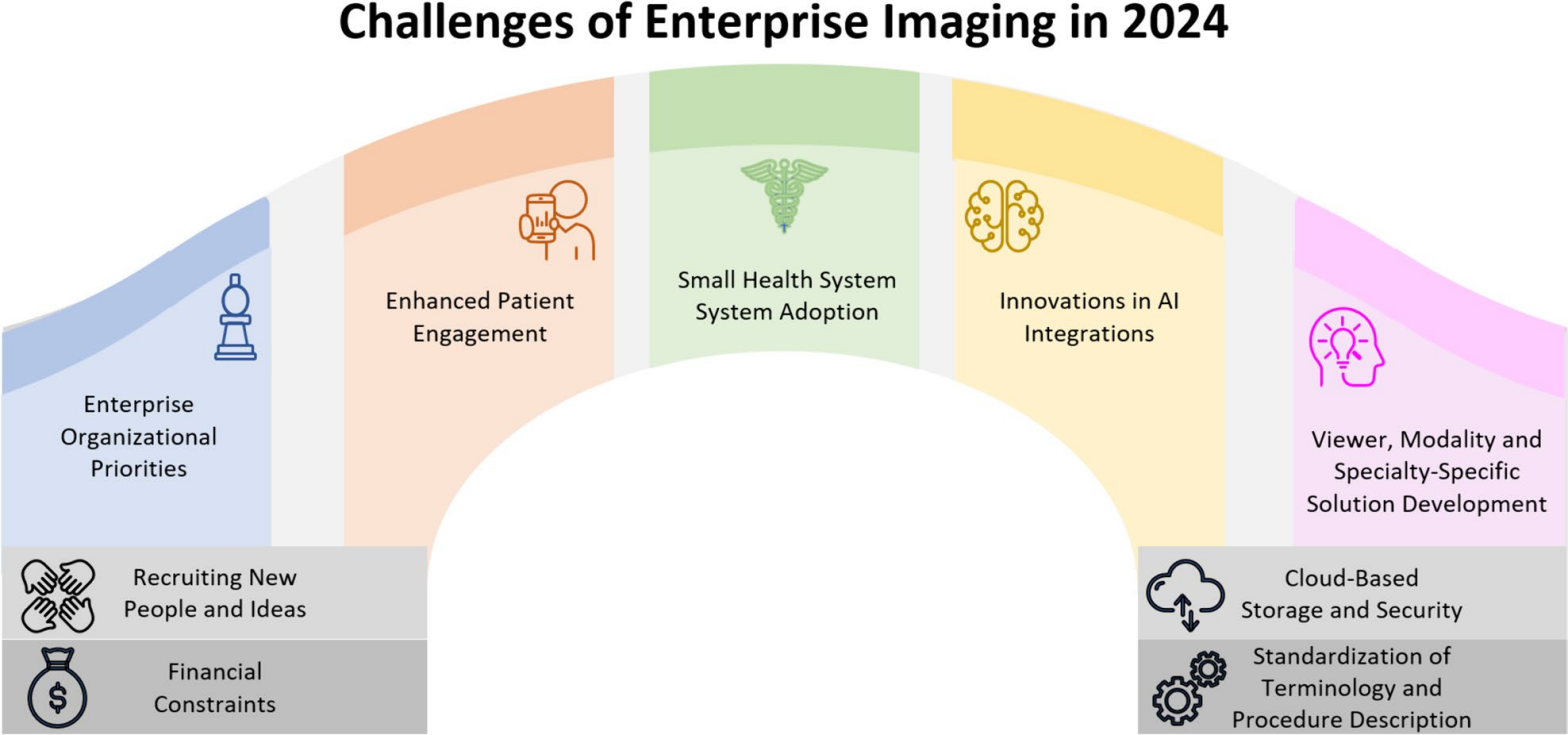
Future challenges for enterprise imaging

With its collective expertise, innovative spirit, collaborative approach, and bias toward action, the HSEIC is poised to continue advancing coordinated, best-practice approaches to imaging across all healthcare organizations. Still, the HSEIC recognizes many challenges remaining (Fig. 3). The following challenges were identified by current and former HSEIC chairs and leaders and are reflective of common enterprise imaging pain points discussed among attendees at the SIIM Annual Meeting.

- Recruiting new people and ideas: The HSEIC has been a lively, volunteer-run organization since its inception. Such organizations require ongoing recruiting efforts to refill their membership with enthusiasm and new ideas. Engaging new imaging experts and informaticists across all medical specialties willing to share their time and experience is vital for the HSEIC’s continued vibrancy. In addition to clinical experts, representatives from imaging technology vendors have been integral to HSEIC, as reflected in its membership and leadership.
- Financial constraints: Healthcare organizations face difficult financial challenges that pressure funding, including negative reimbursement trends, inflation, and a difficult staff recruiting and retention environment. To continue the advancement of enterprise imaging, finding and documenting hospital return on investment (ROI) and return on health (ROH) wherever possible will be important.
- Cloud-based storage and security: The US Office for Civil Rights reports large healthcare breaches rose 93% from 2018 to 2022 [23]. Increasing reports of healthcare cyber incidents impacting care providers, payers, and industry must be considered a call to action. Enterprises will look to the cloud in the right setting for reliable application hosting, scalable data archiving, infrastructure hardware and personnel cost control, and better access control. Nevertheless, cloud migrations require foresight, effort, and experienced imaging vendor partners to maintain or enhance system performance.
- Standardize terminology and procedure description: Developing standardized language and nomenclature across different specialties and procedures is essential for effective communication, data exchange, and data use. Consistent ontology adoption is an overdue, long-term investment. Even if terminology agreement were to occur soon, it would take years for the preferred nomenclature to disseminate substantially into EHRs, modalities, and specialty imaging software. Data elements requiring standardization include body part, procedure description, and medical specialty.
- Enterprise organizational priorities: At the time of the HSEIC’s inception, EHRs and enterprise consolidation were front-of-mind efforts and heavily resourced. Government stimulus money was key in advancing these initiatives. While these initiatives were synergistic for enterprise imaging, many organizations may have since pivoted to competing operational priorities, such as improving patient access, accelerating footprint growth, or enabling competing IT demands, such as artificial intelligence.
- Enhanced patient engagement: Consumerism is increasingly a driver of patient decision-making. Patients are empowered through improved access to their own images and imaging reports and have a better understanding of the costs of their care [24, 25]. More and more patients and families expect a collaborative and highly communicative relationship with their providers [26]. Workflows need to be developed so that patients can better communicate with those providers capturing and interpreting their images. To this end, organizations have worked to image-enable their patient portals [27]. As software advances, patients should also be empowered to upload images obtained at home and initiate the transfer of stored images to another hospital or care provider.
- Small health system solution adoption: In reviewing the advancement of enterprise imaging over the last 10 years, there continues to be a pattern where large health systems are better positioned to implement enterprise imaging than smaller community health systems. This is multifactorial, tied to the difference in governance, professional engagement, financial structure, and technical infrastructure within these different health systems. Larger health systems increasingly have support teams responsible for enterprise imaging; these team members may be distributed within EHR teams, specialty applications informatics support, or may exist as a consolidated enterprise imaging team. Today, HSEIC membership is overweighted toward large enterprises. Since community hospitals represent over 85% of total staffed hospital beds in the USA, this suggests that enterprise imaging still has yet to escape from more niche adoptions [28]. The reasons for these barriers to adoption are several but include compressed operating margins, pluralistic medical staff models in community hospitals, immature imaging workflows in some clinical areas, and the lack of mature technology governance in many community health systems. The role of the voluntary medical staff models should not be underestimated as a barrier, as many community hospitals have multiple private practice groups providing care in specialty areas such as otorhinolaryngology, ophthalmology, and cardiology [29]. In these cases, getting alignment across different competing practices can be as challenging as getting alignment across specialty practices. This challenge of achieving alignment hinders support for enterprise efforts that support multiple service lines.
- Innovations in AI integration: A consolidated enterprise imaging strategy facilitates more efficient data accessibility for developing and monitoring AI models, regardless of specialty. The same data standardization fostered by enterprise imaging workflows also enhances AI data curation and quality, ultimately impacting model accuracy. While many AI ROI and ROH opportunities are available, setting up data pipelines and processes, procuring software, and arranging ongoing monitoring take up enterprise imaging resources and may be unfamiliar territory for IIPs and providers.
- Viewer improvements: Industry partners will continuously pursue more comprehensive patient care by improving their enterprise, advanced, and specialty viewer software to seamlessly integrate images from diverse modalities and specialties. Such progressive tools will regularly bring new application consolidation opportunities. We believe that the next generation of imaging viewers will be able to display imaging across all modalities and all specialties. Additional key features of this next generation of viewers will include the ability to incorporate AI algorithms and their outputs, the ability to view video content (inclusive of sound playback), the ability to view waveforms, and the ability for specialists to annotate images according to the unique needs of their specialty.
- Modality and specialty-specific solution development: ROI for the vendor community is an important driver of system development. Buy-in from industry to develop and enhance enterprise imaging solutions will be critical in supporting the continued adoption and advancement of enterprise imaging solutions. The growth of encounters-based imaging workflow-based point-of-care ultrasound platforms illustrates the benefits of the HSEIC’s clinical and industry partnerships.
  - Sensitive images and privacy: Handling and sharing sensitive images, such as those related to patient identification, sensitive medical conditions, anatomy, or photodocumentation of traumatic injuries, require careful consideration, standardized protocols, and adherence to ethical and privacy standards. Medical photography remains a relatively ungoverned content area in many hospitals with technical and operational risks and difficult-to-implement technical and operational challenges.
  - Ocular imaging: There are unique capture, archiving, and workflow challenges in optometry and ophthalmology clinics that prevent easy integration into an enterprise imaging strategy. While local community eye clinics may have fundoscopic image storage and viewing capabilities, these data typically are disconnected from patient records at prior patient eye clinic locations and hospitals.
  - Pathology imaging: Whole slide pathology is an exciting new domain within imaging informatics. As the pathology specialty becomes more digital, there are opportunities to reinvent traditional clinical workflows and evolve the DICOM standard. Setting up digitizers, expanded networking, cloud storage, viewing applications, and interfacility sharing require extensive resources and specialized subject matter knowledge. Beyond whole slide imaging, there are other forms of visible light pathology images that, over time, should be incorporated into an enterprise imaging infrastructure and processes.
  - Point of care ultrasound (POCUS): Sonographic diagnostic and procedural images are frequently obtained across a healthcare enterprise. These data come from a remarkably heterogeneous modality base. Consistently associating documentation with the ultrasound images to support revenue capture, compliance, risk management, and privileging requires culture change and often significant EHR or dedicated POCUS application build.
  - Video capture: Integrating video content, such as endoscopy or surgical videos, into the enterprise imaging framework and electronic health record while ensuring proper storage, viewing, and sharing capabilities, also requires culture change and technology configuration. New considerations of video editing with partner physicians and patients as consumers in mind may not be a high priority for capturing physicians. Few sites seem to share endoscopic or surgical video with patients consistently well today, even though the 21st Century Cures Act likely requires such electronic health information (EHI) to be shared.

## Conclusion

It is difficult to imagine enterprise imaging without the technical development and community networking the HSEIC facilitates. The community’s 16 (and counting) white papers and IHE profile development leadership have helped to advance the field by identifying challenges and offering real-world workflow solutions. The community’s work has facilitated the integration of emerging modalities such as point-of-care ultrasound, mobile image and video capture, and pathology imaging into the imaging and electronic health record infrastructure. HSEIC has had an eye for the future, attempting to develop standards and address ethical concerns. The community’s future-facing strategy has helped hospitals prepare for new technologies such as artificial intelligence. The leaders of the HSEIC hope the next generation of ambitious imaging informatics experts will build consensus workgroups and partner with industry to address technical challenges while driving adoption locally and globally to improve patient care.

## Data Availability

Not applicable.
